# Dr. S. S. White’s Death

**Published:** 1880-01

**Authors:** 


### DR. S. S. WHITE'S DEATH.

The following from the "*Philadelphia Times*," of December 31st, will carry surprise, sadness and sorrow to the members of the Dental Profession, not only in this country, but throughout the world. From an acquaintance of about thirty years, with Dr. White, I have no hesitancy in endorsing the statement presented in the *Times* concerning him; and it may, in some particulars, be regarded as falling below, rather than overstating the truth. He was much above the common average of men, in the power and balance of his faculties. His judgment, will and perception was naturally of a high order, and had been long under rigid culture. His social qualities were of a high order. As a friend, he was all that is implied by that expression in its strongest import.

The *Times* remarks:

The death of S. S. White, of Philadelphia, which the cable announces took place in Paris yesterday morning, removes from the scene of action, one to whom American dentistry owes more, perhaps, than to any other man, dead or living. His life affords a remarkable example of what energy and perseverance may accomplish, and there are few records in which these attributes are prominent that leave behind them such substantial results. From a penniless boy in an obscure country town, the eldest son of a widow who found a means of subsistance for herself and children, by carrying on business in the confectionery line, in a small way. He rose by his industry and thrift to be the possessor of millions, and as the pioneer in a new era in dentistry to make his name known in almost every country on the habitable globe. He was born in Hulmeville, Bucks County, Pa., in 1822, the son of William R. and Mary White, plain people, with little in the way of worldly possessions, but of eminent respectability. His father died when he was eight years old. The mother, left on her own resources, moved from their little village to Burlington,

New Jersey, where she opened a candy store. The son remained with her, helping her in the business, until he was fourteen years of age, when he came to Philadelphia and entered as an apprentice, the establishment on Vine street, of his uncle, S. Wesley Stockton, dentist and manufacturer of artificial teeth. That was forty-three years ago. Samuel Stockton White learned his trade, and as soon as he came of age, began to practice dentistry in his uncle's office, in addition to superintending his manufactory. Shortly afterward he removed to Eighth street above Race, and went into practice for himself. There he remained till 1845. In that year, he took in two partners—Asahel Jones of New York, and John R. McCurdy, of Philadelphia. The manufacture of teeth began to make up a large part of their business, and in 1846, Mr. White seeing a wider field of operations, relinquished his practice of dentistry and began to devote his time wholly to making teeth and dental instruments. They remained on Race street till 1848. Then they removed to a property on Arch street, below Sixth, which they had purchased and fitted up to accommodate their increasing business. Soon, the business became the leading one of the country. White's teeth and White's dental instruments began to be known everywhere. Branch houses were established in New York, Boston and Chicago. Finally the partners sold out to their senior in the firm. McCurdy retired in 1859, Mr. White purchasing his interest, including real estate, for $140,000. Jones retired in 1861, his interest, including real estate, bringing exactly the same amount.

The business had now grown to stupendous proportions. White's dental supplies began to be known all over the world. Every invention that came out was bought up by the ambitious manufacturer and made use of one way or another. In 1867 he removed again, and for the last time, from Arch street down to the massive building on the southeast corner of Twelfth and Chestnut streets, the lower portion of which is occupied by Bailey, Banks & Biddle's jewelry store. This is one of the largest buildings in the city, as well as among the costliest. It is five stories high, runs back to Sansom street and cost half a million dollars. Here the work of manufacturing teeth and dental instruments has been going on ever since. Over 300 hands are employed and 4,000,000 teeth are turned out annually.

A prominent dentist, speaking of the death of Dr. White yesterday, said American dentistry owed him more than it did to any other ten men living.

"It may be said to date its beginning with his entrance into business as a manufacturer of teeth and dental instruments. When he began this business dentistry was in its infancy. The porcelain teeth which up to that time had been placed upon the market were but wretched imitations of nature and looked like so many split beans. Dr. White's first efforts were directed toward improving their shape, translucency and adaptability to the mouth. He then devoted himself to the work of reproducing in form and style the varieties of teeth found in persons of different temperaments, so that the practicing dentist might be able to find in his stock teeth conforming in their peculiarities to youth and age, male and female, blondes and brunettes and all the diversified types of humanity. In these efforts he has succeeded to an extent that would seem to leave nothing more to be desired. He has received premiums from all the leading industrial exhibitions of the world, including medals from each of the great world's fairs of London, Paris, Vienna, Chili and New York, amounting in all to seventy-five medals, the grand medal of honor from Vienna—of which testimonials only eight were received by American exhibitors—inclusive."

In addition to the remarkable success attending him in the manufacture of teeth he has been equally successful in the manufacture of dental supplies. From the smallest hand instrument for separating the teeth or excavating for the purpose of filling to the latest improved style of dentists' chair, which he is said to have taken great pride in, his manufactory has become noted. It is mentioned as a somewhat remarkable fact that the various hand instruments known as scalers, excavators, pluggers and the like made in his establishment have found their largest market in foreign countries which are supposed to excel in the manufacture of the finest specimens of edge tools.

But it was not alone to the manufacture of implements and appliances that his energies have been devoted. For thirty-three years he has exercised what may be termed a controlling influence in dental literature--first in his connection with the *Dental News-Letter*, a quarterly journal, which was published for twelve years, and then succeeded by the *Dental Cosmos*, now in its twenty-second volume.

Dr. White left home, in company with a son and nephew, in November last, to travel for a time in Europe for the benefit of his health. They had been in Paris but a few days till he was taken suddenly ill, and this was followed within forty-eight hours with the news of his death. Congestion of the brain was the cause.

Any sketch about him without a mention of his readiness to lend a hand to the cause of invention in directions other than his own profession would be in complete. He became interested in the development of the Gray telephone as early as 1875, and by advancing money to the inventor was in a measure instrumental in bringing about the present satisfactory system of telephonic operations. He was also a stockholder in the American Speaking Telephone Company, comprising the Phelps and Gray patents.

Dr. White was, for many years, a member of the Arch Street M. E. Church. He was also a member of the Union League, the Reform Club, the Franklin Institute, the American Association for the Advancement of Science, the United States Board of Trade, the Pennsylvania Association of Dental Surgeons and of many other business, social and benevolent organizations. He dies worth about one and a half millions.

